# The *C. elegans* OCTR-1 and Human Alpha-2A Adrenergic Receptors are Methylated within the Third Intracellular Loop by Human PRMT5 *in vitro*

**DOI:** 10.17912/micropub.biology.000546

**Published:** 2022-03-23

**Authors:** Alexander Bowitch, Tyler M. Chinsky, Michael C. Yu, Denise M. Ferkey

**Affiliations:** 1 Department of Biological Sciences, University at Buffalo, The State University of New York, Buffalo, NY 14260 USA

## Abstract

Arginines within the third intracellular loop of the *C. elegans* OCTR-1 and human ADRA2A receptors are methylated by the human protein arginine methyltransferase PRMT5 *in vitro* . Methylation of these residues could serve to modulate receptor signaling *in vivo* .

**Figure 1. Human PRMT5 methylates the third intracellular loop of the C. elegans OCTR-1 receptor and the human alpha-2A adrenergic receptor (ADRA2A) in vitro .**
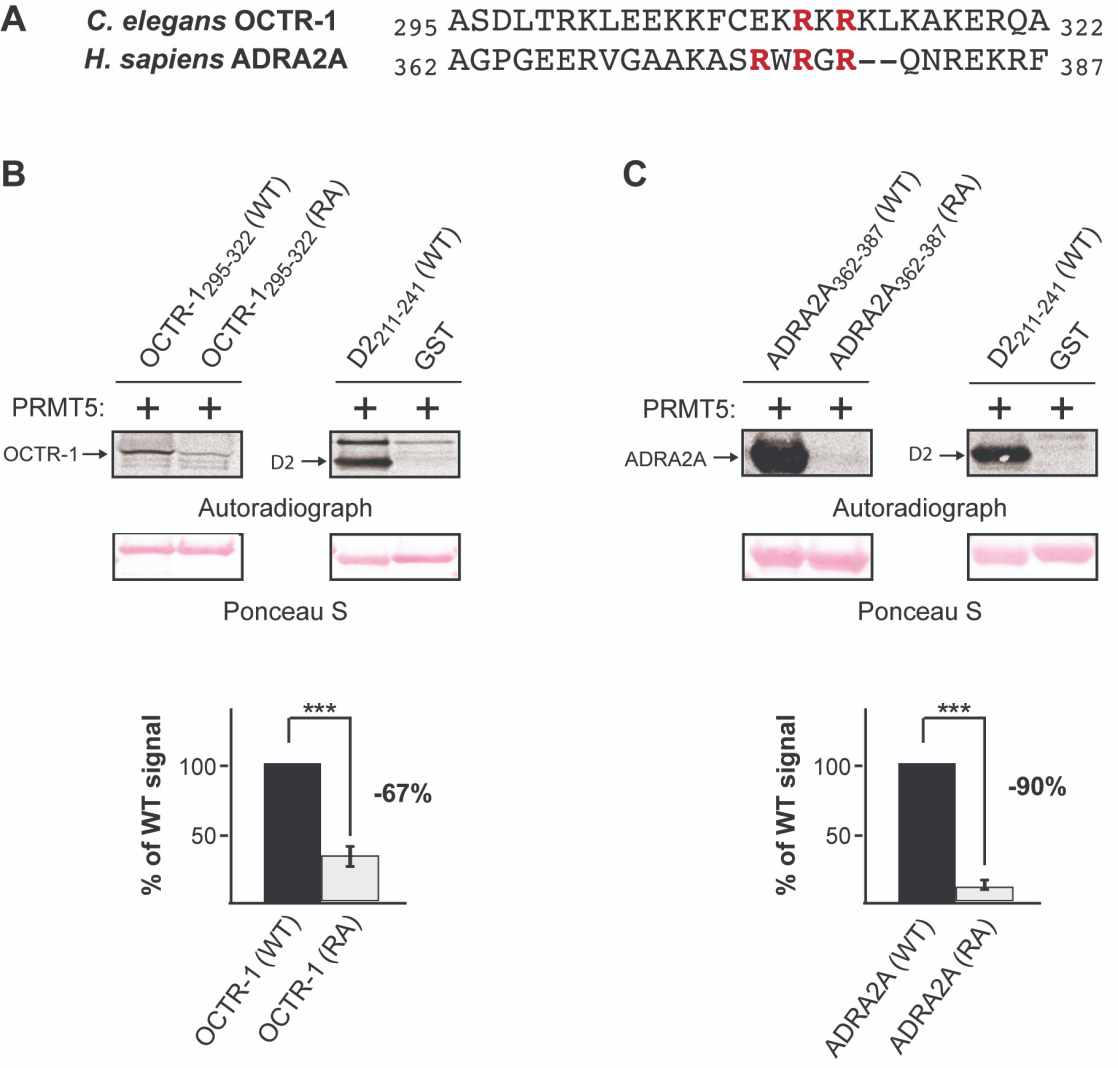
**(A)** Alignment showing conservation of the predicted arginine methylation motifs in the *C. elegans* OCTR-1 and human ADRA2A receptors. The entire third intracellular loop (3 ^rd^ ICL) of OCTR-1 is predicted to have ~121 amino acids, representing residues 203-323 [SWISS-MODEL, based on (Qu *et al.* 2020)]; only residues 295-322 of the 3 ^rd^ ICL are shown and aligned with the corresponding residues of the 3 ^rd^ ICL of ADRA2A. Arginines within the predicted methylation motifs (RXR) are shown in red. **(B and C)** Representative blots for the *in vitro* methylation assays. A wild-type (WT) and mutant (RA) recombinant fragment of the 3 ^rd^ ICL of OCTR-1 (residues 295-322) and ADRA2A (residues 362-387), flanked both amino- and carboxy-terminally with an S-tag to increase solubility, and fused to glutathione S-transferase (GST) were used in an *in vitro* methylation assay with active recombinant human PRMT5. There are no arginines within the S-tag. A fragment of the human D2 dopamine receptor was used as the positive control for methylation (Likhite *et al.* 2015) and GST alone served as the negative control. Top: Autoradiographs show that WT OCTR-1 and ADRA2A are methylated by PRMT5. Fragments with the conserved arginines (shown in red, Figure 1A) changed to alanines were not efficiently methylated. Ponceau S staining of the polyvinylidene difluoride (PVDF) membranes was performed to demonstrate equivalent loading of the WT and RA receptor fragments. Bottom: Quantification of the degree of methylation of the receptor fragments based on densitometric analysis of the autoradiographs. Loss of the methylation motif resulted in a 67% decrease in OCTR-1 methylation and a 90% decrease in ADRA2A methylation. Data are means, +/- the standard error of the mean (SEM) from three independent experiments. *** = p < 0.001

## Description

One way that G protein-coupled signal transduction is regulated intracellularly is through post-translational modification of the receptors (G protein-coupled receptors; GPCRs). While phosphorylation remains the best studied of these modifications (Komolov and Benovic 2018), a proteomics analysis previously identified several GPCRs as substrates of an anti-methylarginine antibody (Boisvert *et al.* 2003). We subsequently reported the first evidence that GPCRs can be functionally regulated by protein arginine methylation: D2-like dopamine receptors (human D2 and *C. elegans* DOP-3 receptors) (Likhite *et al.* 2015) and the *C. elegans* SER-2 tyramine receptor (Bowitch *et al.* 2018). In each case, conserved arginines within the third intracellular loop (3 ^rd^ ICL) were methylated by PRMT5 *in vitro* , and experiments examining signaling in cell culture (D2) and behavior in *C. elegans* (DOP-3 and SER-2) suggested that arginine methylation serves to promote receptor signaling (Likhite *et al.* 2015; Bowitch *et al.* 2018). However, the mechanism by which it does so is not yet known. In the case of D2, methylation may alter interaction with regulatory proteins that bind to the region of the 3 ^rd^ ICL that contains the methylated arginines (Bowitch *et al.* 2021).

In a bioinformatics analysis, Likhite *et al.* (2015) identified 300 human GPCRs that contained an intracellular putative methylation motif [RGG or RXR, where X can be any amino acid (Bedford and Clarke 2009; Thandapani *et al.* 2013)]. Nearly 70% of these motifs were conserved in the corresponding mouse and rat receptors, and ~1% (seven) were conserved in *C. elegans* (Likhite *et al.* 2015). Among these, the *C. elegans* OCTR-1 octopamine receptor was identified to contain a putative methylation motif that is conserved in the human alpha-2A adrenergic receptor (ADRA2A) (Likhite *et al.* 2015). Octopamine was first discovered in the salivary glands of the octopus in 1948 (Juorio and Molinoff 1974), and has since been determined to be a biogenic amine in invertebrates that functions as a neurotransmitter, neurohormone and neuromodulator (Scheiner *et al.* 2002). It is considered the invertebrate counterpart of adrenaline due to their structural and receptor similarity (Roeder 1999). Adrenaline and noradrenaline (with a lower potency) are endogenous ligands of ADRA2A (Altosaar *et al.* 2021).

We wished to determine if the predicted methylation motif within the 3 ^rd^ ICL of OCTR-1 and ADRA2A (Figure 1A) could serve as a substrate for human PRMT5 *in vitro* . A recombinant fragment of the 3 ^rd^ ICL of each (see Figure Legend for details) was methylated by PRMT5 (Figure 1B, 1C). To determine whether the arginines of the predicted methylation motif of OCTR-1 (Arg ^311^ and Arg ^313^ ) were necessary for methylation, we generated a recombinant fragment in which these arginines were changed to alanine [OCTR-1(RA)]. Methylation of this fragment was decreased 67% compared to the wild-type (WT) fragment (Figure 1B). Similarly, to determine whether the arginines of the predicted methylation motif of ADRA2A (Arg ^376^ , Arg ^378^ and Arg ^380^ ) were necessary for methylation, we generated a recombinant fragment in which these arginines were changed to alanine [ADRA2A(RA)]. Methylation of this fragment was decreased 90% compared to the WT fragment (Figure 1C). Both peptides contain additional arginines that are not part of described PRMT recognition motifs. Context-dependent methylation of these could account for the residual signals, as PRMT5 is not known to methylate lysines. These data establish the 3 ^rd^ ICL of both receptors as substrates for PRMT5 *in vitro* , and suggest that the highlighted arginines are key sites of methylation within this region.

GPCRs are the largest family of cell surface receptors and are involved in diverse physiological processes. Accordingly, mutations that disrupt GPCR signaling can result in many different diseases (Schöneberg *et al.* 2004; Zalewska *et al.* 2014). Nearly 35% of all pharmaceuticals target GPCRs in an attempt to therapeutically control their signaling (Sriram and Insel 2018). Notably, arginine methylation appears to have a modulatory effect on GPCR signaling (Likhite *et al.* 2015; Bowitch *et al.* 2018). Thus, identifying additional receptors that can be methylated provides new potential targets for treatments based on regulating or altering GPCR methylation status.

## Methods

Protein purification

The following GPCR third intracellular loop fragments were purified using the previously described protocol (Bowitch *et al.* 2018):

GST::S-tag::OCTR-1 _295-322_ ::S-tag

GST::S-tag::OCTR-1 _295-322_ (R311A/R313A)::S-tag

GST::S-tag::ADRA2A _362-387_ ::S-tag

GST::S-tag::ADRA2A _362-387_ (R376A/R378A/R380A)::S-tag

The S-tag sequence (KETAAAKFERQHMDS) was added to aid in protein solubility and does not contain any arginines.

GST and GST::D2 _211-241_ were expressed and purified as previously described (Likhite *et al.* 2015).

*In vitro* methylation

The *in vitro* methylation assay was performed similarly to as described (Likhite *et al.* 2015; Bowitch *et al.* 2018) in a total volume of 20 µl with 4 µg of substrate, 1 µl of recombinant human PRMT5+MEP50 protein (Abcam; catalog number 271720), and 2 µCi of S-[methyl- ^3^ H]adenosyl-L-methionine (55 to 85 Ci/mmol; PerkinElmer) in 20 mM phosphate buffer (pH 7.4). Reactions were incubated at 30°C for 4 hours, resolved by Bolt 4-20% SDS-polyacrylamide gel electrophoresis (Thermo-Fisher), and then transferred to polyvinylidene difluoride (PVDF) membranes. The membranes were stained with Ponceau S, dried, and exposed to a low energy phosphorimager screen for at least two weeks and subsequently developed using an Azure Sapphire Biomolecular Imager. Band intensities were quantified with Bio-Rad ImageLab software (6.1.0 build 7) and were normalized according to gel loading (Ponceau S stained bands quantified by ImageJ, version 1.53n). The Student’s one-tailed t-Test was used for statistical analysis.
